# Evaluation of Cumulative Effect of Standard Triple Immunosuppression on Prevention of De Novo Donor Specific Antibodies (dnDSA) Production in Children after Kidney Transplantation—A Retrospective and Prospective Study

**DOI:** 10.3390/children8121162

**Published:** 2021-12-09

**Authors:** Agnieszka Urzykowska, Barbara Piątosa, Urszula Grycuk, Grzegorz Kowalewski, Zbigniew Kułaga, Ryszard Grenda

**Affiliations:** 1 Department of Nephrology, Kidney Transplantation & Hypertension, Children’s Memorial Health Institute, 04-730 Warsaw, Poland; a.urzykowska@ipczd.pl; 2 Histocompatibility Laboratory, Children’s Memorial Health Institute, 04-730 Warsaw, Poland; b.piatosa@ipczd.pl (B.P.); u.grycuk@ipczd.pl (U.G.); 3 Department of Surgery and Organ Transplantation, Children’s Memorial Health Institute, 04-730 Warsaw, Poland; g.kowalewski@ipczd.pl; 4 Department of Public Health, Children’s Memorial Health Institute, 04-730 Warsaw, Poland; z.kulaga@ipczd.pl

**Keywords:** Vasudev score, cumulative strength of immunosuppression, TAC variability and concentration, dnDSA, kidney transplantation

## Abstract

De novo Donor Specific Antibodies (dnDSA) are associated with inferior graft outcomes. Standard immunosuppression is expected to prevent dnDSA production in low-risk patients. We have evaluated a cumulative effect of a triple immunosuppression (CNI/MMF/Pred), as well as TAC concentration and coefficient of variation on the incidence of dnDSA production. Overall, 67 transplanted patients were evaluated in retrospective (dnDSA for-cause; *n* = 29) and prospective (dnDSA by protocol; *n* = 38) groups. In the retrospective group, the eGFR value at first dnDSA detection (median interval—4.0 years post-transplant) was 41 mL/min/1.73 m^2^; 55% of patients presented biopsy-proven cAMR, and 41% lost the graft within next 2.4 years. Patients from the prospective group presented 97% graft survival and eGFR of 76 mL/min/1.73 m^2^ at 2 years follow-up, an overall incidence of 21% of dnDSA and 18% of acute (T cell) rejection. None of the patients from the prospective group developed cAMR. Median value of Vasudev score within 2 years of follow-up was not significantly higher in dsDSA negative patients, while median value of TAC C_0_ > 1–24 months post-transplant was 7.9 in dnDSA negative vs. 7.1 ng/mL in dnDSA positive patients (*p* = 0.008). Conclusion: dnDSA-negative patients presented a higher exposure to tacrolimus, while not to the combined immunosuppression.

## 1. Introduction

The development of de novo Donor Specific Antibodies (dnDSA) is a recognized risk factor of antibody mediated rejection and inferior outcome in kidney transplantation [1,2,3,4,5].

Although regular testing for dnDSA has been recommended in kidney transplantation, consensus on optimal timing and methodology (prospective screening vs. in-case monitoring, i.e., initiated in graft dysfunction) has not been reached [6,7]. Inadequate immunosuppression is considered as one of the several identified risk factors of dnDSA production; however, the relevance of individual immunosuppressive drugs in terms of this risk has not been fully clarified [8,9]. Currently, most of European pediatric kidney transplant recipients with low-to-moderate immunological risk receive standard triple maintenance protocol (CNI/MMF/Pred) without induction [10]. Evaluation of exposure to basic immunosuppressive drugs is based on Therapeutic Drug Monitoring (TDM). Regular assessment of tacrolimus (TAC), cyclosporine A (CsA), sirolimus (SIR), and mycophenolic acid (MPA) through (C_0_) blood concentrations is used in common practice, while evaluation of the area under the curve (AUC) is used in more complicated cases [11,12,13]. Several parameters and key endpoints of kidney transplantation evaluated in clinical practice and trials, such as renal function, incidence of rejection, and graft survival, are adjusted to TDM-related parameters of a single drug, predominantly one of calcineurin inhibitors (usually TAC), regarded as the cornerstone immunosuppressant [11,14]. However, most patients are simultaneously exposed to three drugs in the triple immunosuppressive protocol (CNI + MMF + Pred). Therefore, final outcomes may be affected by the cumulative drug exposure. Vasudev and co-workers have developed an original immunosuppressive score to present in form of a numeric value the combined strength of common drugs used in adult patients [15]. The modification of this score for pediatric transplant patients developed by Höcker et al. has been based on an adjustment of the relevant drug-related adult scores to the body surface area (as presented in Table 1) [16].

The suboptimal range of C_0_ TAC blood concentration in long-term follow-up has been identified as one of risk factors relevant for dnDSA production. Data from studies in living-related adult transplant patients treated with the extended-releas e tacrolimus (combined with various drugs in a triple maintenance protocol) demonstrated significant difference between mean C_0_ TAC values in the dnDSA-positive and -negative patients (4.88 vs. 3.69; *p* = 0.023) [17]. Blood concentration of the regular immunosuppressive drugs has been reported as an important risk factor also in pediatric transplant patients [12,18]. High intra-patient coefficient of variation (>30%) of TAC C_0_, which is also considered a surrogate marker of non-adherence in adolescents and young adult patients, has been identified as a significant risk factor of dnDSA development [19,20,21,22]. We have hypothesized, that the incidence of dnDSA production is associated not only with inadequate TAC C_0_ and a high intra-patient variability of TAC concentration, but also with the suboptimal value of the cumulative immunosuppressive load of the standard triple immunosuppression protocol. To verify this hypothesis, we conducted a single-center study that included a retrospective arm, with dnDSA evaluation based on clinical indication (in-cause) and a prospective arm, with regular dnDSA (by protocol) monitoring.

## 2. Materials and Methods

**Figure 1:**
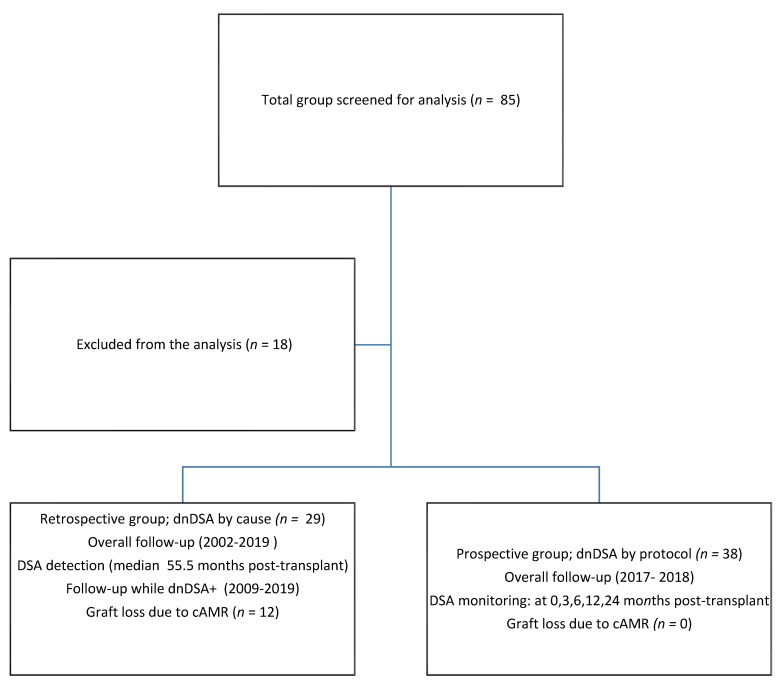
Study flowchart.

Low to moderate immunological risk and triple protocol of immunosuppression were criteria of inclusion to the study group. Overall, 85 patients were preliminarily screened, including 29 in the retrospective group (median age of 8.1 years), and 38 in the prospective group (median age of 11.4 years); however, 18 patients were excluded from the analysis due to pre-transplant presence of DSA and/or further loss to follow-up. The prospective group included 38 patients after first and one patient (2.6%) after second transplantation, while the retrospective group included 29 patients after first and two patients (6.8%) after second kidney transplantation. The vast majority of patients received a combination of TAC (86.2% in the retrospective vs. 81.6% in the prospective group), MMF (86.2% in the retrospective vs. 97.4% in the prospective group), and Pred (100% in both groups). More than 3/6 HLA mismatches were identified in 23 patients (60%) from the prospective group and 17 patients (58%) from the retrospective group. Cumulative HLA A + B + DR mismatch was similar in both groups (median 4 vs. 4; *p* = 0.3; Mann-Whitney test). The flowchart of the study is presented on Figure 1 and baseline characteristics of the enrolled patients are presented in Table 2.

The therapeutic range of blood CNI concentration beyond six months post-transplant was set at 5–10 ng/mL for TAC C_0_ and 100–150 ng/mL for CsA C_0_.

MMF was administered at 600 mg/m^2^/b.i.d. dose, tapered to 300 mg/m^2^ beyond the first month post-transplant in TAC treated patients.

Maintenance prednisone was administered at 60-30-15-9-6 mg/m^2^ doses, tapered in one-week intervals, to reach the final dose below 0.1 mg/kg/day.

Analysis of associations between the degree of immunosuppression and the incidence of dnDSA was performed in a prospective group.

eGFR was calculated according to modified Schwartz formula [23].

The in-cause evaluation of dnDSA (in the retrospective group) was performed in cases of deterioration of kidney function, expressed as an increase of serum creatinine concentration by ≥30% from baseline [24] in two consecutive evaluations, after exclusion of relevant clinical reasons, such as urinary tract obstruction/infection or high blood concentration of CNI.

Analysis of a long-term kidney function, in terms of probability of chronic eGFR decrease ≥30% from baseline (eGFR at 1 month after transplantation) during 24 months of follow-up was performed in a prospective group.

All sera were screened for the presence of IgG anti-HLA antibodies using the Labscreen Mixed assay (One Lambda, Canoga Park, CA, USA) and the Luminex bead flow method at established follow-up intervals. Sera found to contain HLA antibodies underwent further testing to assign specificity of the target antigen(s) using One Lambda Labscreen Single anti-Class I and/or anti-Class II kits. All tests were performed in accordance with manufacturer’s instructions [25,26]. Protocol screening for presence of dnDSA was performed in all consecutive patients in the prospective group, irrespective from the current graft function, directly before kidney transplantation, and at 3, 6, 12, and 24 months after kidney transplantation.

Pathologic diagnosis of cAMR was based on updated Banff criteria [27]. Therapeutic regimens used in the episodes of rejection were adjusted to the biopsy-proven pathologic pattern and the concomitant presence/absence of dnDSA. Patients with cellular rejection were treated with 3–6 doses of 10 mg/kg methylprednisolone, and/or thymoglobulin (starting dose of 1.25–1.5 mg/kg, then adjusted to the T_CD3_ count < 50 μL in steroid-resistant cases), while patients with cAMR were treated with combination of plasmapheresis, IVIG (2 g/kg), and rituximab (1 dose 375 mg/m^2^) [28].

### Statistical Analysis

Statistical analyses were carried out with statistical software SAS (SAS version 9.4; SAS Institute Inc., Cary, NC, USA). All continuous variables were checked for normal distribution using a Kolmogorov–Smirnov test. Descriptive statistics provided medians with interquartile ranges (IQR) in case of continuous variables that did not meet normal distribution criteria, and proportions for categorical variables. Data analysis was performed using a Mann–Whitney U test (for quantitative data with non-normal distribution), categorical variables were compared among groups using chi-squared and Fisher’s exact test. Linear mixed model [29] was applied to compare repeated tacrolimus concentrations and Vasudev scores between groups. Normality of residuals was checked by visual inspection of QQ plots. A Kaplan–Meier estimate was used to analyze the probability to maintain kidney function over time. Log-rank analysis was used to evaluate the survival curves. The Cox proportional-hazards model was used to analyze the risk of a significant decrease of baseline eGFR value (>30% from baseline).

The study was conducted in accordance with the principles of Helsinki Declaration and after an approval of the Ethical Committee at the Children’s Memorial Health Institute (Warsaw, Poland) (no. 3/KBE/2017). Informed consent was received from all relevant patients and all legal guardians. The project was partially funded by the institutional research grant no. S/150 (Children’s Memorial Health Institute, Warsaw, Poland).

## 3. Results

**Figure 2:**
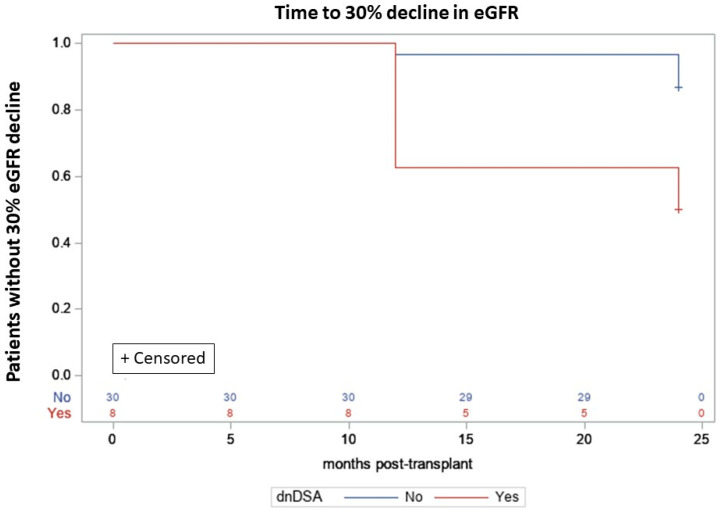
Probability of the decrease of eGFR < 30% from baseline in the dnDSA- positive and the dnDSA- negative patients from a prospective group, log rank *p* = 0.0129.

The overall incidence of dnDSA was 100% in the retrospective and 21% in the prospective group (*p* < 0.0001). The incidence of dnDSA was regularly increasing in a prospective group with time after transplantation. It was 8% at 3 months, 11% at 6 months, 16% at 14 months, and finally 21% after 2 years of follow-up. Median eGFR value at detection of dnDSA was 41 in the retrospective and 85 mL/min/1.73m^2^ in the prospective group (*p* = 0.004). The presence of dnDSA in a prospective group was associated with higher risk of a further eGFR decrease ≥30% from baseline (adjusted hazard ratio [aHR] 4.37; 95% CI, 1.058–18.038; *p* = 0.0415), in the Cox regression model, which was adjusted for age at transplant, baseline eGRF (one month after transplant), and HLA A + B + DR mismatches. The Kaplan–Meier curves illustrating this effect are presented in Figure 2.

The incidence of a biopsy proven rejection was 66% in the retrospective vs. 18% in the prospective group (*p* = 0.0001), while the incidence of graft loss in cAMR was 41% vs. 0 (*p* < 0.0001). These data are summarized in Table 3.

All but one episode of acute cellular rejection diagnosed in the patients from the prospective group have been successfully treated. The remaining (one) patient has maintained the graft function, but with eGFR of <15 mL/min/1.73 m^2^. The efficacy of cAMR treatment (in retrospective group only) was limited, as the relevant incidence of a graft loss (due to cAMR) was 41%.

Analysis of associations between the degree of maintenance immunosuppression and the incidence of dnDSA in a prospective group showed that the median values of tacrolimus concentration (TAC C_0_) in all consecutive evaluations between months 1 and 24 after transplantation were significantly higher in the dnDSA -negative patients (7.9 vs. 7.1 ng/mL; *p* = 0.088). The value of a coefficient variation of TAC C_0_ was not significantly different between dnDSA -negative and dnDSA- positive patients (31 vs. 29; *p* = 0.56). The data are presented in Table 4.

The analysis of the cumulative Vasudev scores did not show a significant difference between dnDSA = negative and dnDSA = positive patients from a prospective group, at any time point after transplantation. The data are presented in Table 5.

### Summary of Results

- The incidence of dnDSA was regularly increasing in a prospective group (DSA screening by protocol) with time after transplantation and was 8% at 3 months, 11% at 6, 16% at 14 and finally reached 21% after 2 years of follow-up;
- The presence of the dnDSA in patients from the prospective group, was associated with a >4.5 higher risk of inferior graft function expressed as a chronic decrease of eGFR by ≥30% from baseline within 2-year follow-up;
- A median value of all consecutive evaluations (from >1 to 24 months after transplantation) of blood TAC C_0_ was in the dnDSA- negative patients significantly higher than in the dnDSA- positive patients in a prospective group (7.9 ng/mL vs. 7.1 ng/mL; *p* = 0.0088);
- dsDSA- positive and the dnDSA -negative patients did not differ significantly in terms of TAC C_0_ variability, evaluated during 2-year follow-up;
- Vasudev score was not significantly different between the dnDSA-seronegative and dnDSA- positive patients at 1, 12 and 24 months after transplantation.

## 4. Discussion

Overall, the results of this study support the previously published data regarding the critical impact of dsDSA appearance on the risk of humoral rejection and deterioration of kidney function in pediatric recipients [1,2,5,7]. The retrospective and prospective parts of this report demonstrate two different sides of the clinical problem related to production of dnDSA. In the retrospective setting, the testing for dnDSA was limited to clinically indicated cases and aimed at verification of the cause of kidney function deterioration (expressed as an increase of serum creatinine by ≥30% from a baseline in two consecutive evaluations). The confirmed incidence of dnDSA in this group was 100%, and was associated with biopsy-proven cAMR in 66% cases, as well as with further inferior outcome expressed as death-censored graft loss within next 2.4 years in 41% of cases. Long-term follow-up from transplantation to first for-cause detection (by cause) of dsDSA and low eGFR (41 mL/min/1.73 m^2^) suggested that relevant prospective screening performed after transplantation may possibly reveal much earlier the presence of dnDSA and encourage modification of immunosuppression. The prospective arm of the study, with consecutive patients on a triple maintenance immunosuppression screened for dnDSA presence by protocol up to two years after transplantation, demonstrated an overall incidence of 21% of the dnDSA generation. The incidence was regularly increasing with time after transplantation, from 8% at 3 months, 11% at 6 months, and 16% at 12 months to 21% after 2 years. This was accompanied by a relatively low incidence (18%) of biopsy proven T-cell-mediated acute rejection. None of the patients from the prospective study group demonstrated features of cAMR in a kidney biopsy, and this was associated with 100% one-year and 97% 2-year graft survival. The clinical relevance of the timing of dsDSA occurrence was discussed in different reports. Lee et al. have reported that patients developing dnDSA < 1 year after transplantation presented worse outcomes in terms of 10-year graft survival compared with patient with late dnDSA developers (27 vs. 80%) [30]. Cioni et al. compared the outcomes between early-onset (dnDSA < 1-year post-transplant) and late-onset (dnDSA > 1-year post-transplant) groups of patients, presenting comparable profile of risk factors. They did not show a significant difference in terms of cAMR and graft loss in 5-year follow-up between early- and late- dnDSA [31]. Engen et al. reported the lack of correlation between generation of dnDSA and duration of time to the >30% decline in eGFR or graft loss (adjusted hazard ratio, 0.88; *p* = 0.598). In his study, the presence of dnDSA detected in the for-cause testing was associated with a 2.8 times increased risk of decline in graft function (*p* = 0.034) and 7.34 times increased risk of graft loss (*p* = 0.020), as compared with the patients who did not develop dnDSA [7]. In our study, the association between appearance of dnDSA in patients screened for protocol and further risk of deterioration of kidney graft function by ≥30% was significantly higher in the dnDSA- positive patients (aHR 4.37; 95% CI, 1.058–18.038; *p* = 0.0415). Results of our study demonstrated, that in clinical practice patients presenting dnDSA in a prospective manner had lower degree of the post-transplant immunosuppression, expressed mainly as a lower exposure to the main drug, tacrolimus (TAC). The cumulative effect of all three immunosuppressive drugs included in a protocol, expressed as Vasudev score, was not a significant factor. Despite a long history of tacrolimus use in kidney transplantation, the optimal range of TAC blood concentration beyond three months after transplantation is still discussed. Considering data from available reports, the routinely used wide range of 5 to 10 ng/mL should be probably narrowed on individual basis. Hiramitsu et al., who evaluated the impact of TAC C_0_ on prevention of dnDSA production in adult living-related kidney transplant recipients, reported that mean TAC C_0_ concentration was significantly higher in the dnDSA negative than in the dnDSA positive patients receiving triple-drug immunosuppressive protocol (4.60 ng/mL, interquartile 4.05–5.14 vs. 3.85 ng/mL, interquartile 3.53–4.18; *p* = 0.001. The authors also compared the trough concentration of MPA (mycophenolic acid), and found no difference between two groups, which suggested the major role of TAC in preventing the dnDSA production [17]. Davis et al. have evaluated the impact of mean TAC C_0_ on the risk of dnDSA in a cohort of 538 adult patients during the first year after kidney transplantation, and found that mean TAC C_0_ < 8 ng/mL was associated with dnDSA generation at 6 months (odds ratio [OR] 2.51, 95% CI, 1.32–4.79, *p* = 0.005) and 12 months (OR 2.32, 95% CI. 1.30–4.15, *p* = 0.004), with graded increase in risk correlating with lower mean TAC C_0_ [32]. In order to avoid the effect of the post-transplant timing of the drug evaluation on results, we have compared the median values of all consecutive TAC C_0_ concentrations, starting from the first to the 24th month after transplantation in a prospective group. We found that the median value of consecutive TAC C_0_ in patients, who developed dsDSA was significantly lower (7.1 vs. 7.9 ng/mL; *p* < 0.0088). Beland et al. analyzed the clinical outcome in a group of 42 non-sensitized patients who developed dnDSA. The authors found an inverse correlation between mean tacrolimus concentration and risk of graft loss (HR, 0.49; 95% CI, 0.33–0.75; *p* = 0.001), demonstrating that higher tacrolimus concentration in patients with dnDSA was associated with better kidney graft survival. Mean TAC C_0_ below 5.3 ng/mL in the first two years post-dnDSA detection was found to be a strong, independent predictor of graft loss [33]. Gatault at al., who evaluated the clinical relevance of a TAC C_0_ threshold within the first year after transplantation in terms of prevention of dnDSA production and acute rejection in adult patients of low immunological risk have found that maintaining TAC C_0_ > 7 ng/mL during the first year post-transplant prevented the development of dnDSA [34]. Some reports demonstrate that the value of TAC concentration measured as early as one month after transplantation is an independent factor of later outcome. Yin et al. have evaluated clinical data of 1415 patients after living-related kidney transplantation assigned to three groups depending on TAC C_0_ level: low (410 patients, tacrolimus trough level < 5.35 ng/mL in the first month), median (466 patients, tacrolimus trough level 5.35–7.15 ng/mL), and high-level group (539 patients, tacrolimus trough level > 7.15 ng/mL). Patients from the median-level group demonstrated a lower risk of AR than patients from the low-level group, (AR, 12.4% versus 5.7%, *p* = 0.02) [35]. Therefore, with respect to the commonly used protocols, maintaining TAC C_0_ beyond three months post-transplant within the range of 5–10 ng/mL, we suggest that the lower value in this range should markedly exceed 7 ng/mL in order to increase the efficacy of prevention of the dnDSA production. In contrast to other reports, we have observed a non-significant difference of TAC C_0_ variability (29% vs. 31%; *p* = 0.56) between dsDSA-positive and dnDSA-negative patients. Aksoy et al. showed, that the tacrolimus CV 32% cutoff value was the most accurate measure to identify dnDSA development in 67 pediatric kidney transplant recipients (AUC 0.713). The development of dnDSA during follow- up period was associated with a higher percentage of patients with a tacrolimus CV > 32% between 6 and 12 months, and over 1 year after transplantation (67% vs. 31% and 83% vs. 47%, respectively) [20]. In a pediatric study including 38 patients, Solomon et al. demonstrated that every 10% increase of a tacrolimus variability was associated with a 53% increase of the odds ratio for developing de novo DSA (*p* = 0.048, 95%CI, 1.0005, 1.11). Higher tacrolimus variability was associated with an increased incidence of allograft rejection at CV cut-off point ≥ 30%, (0% vs. 42%, <30 and ≥30%, respectively, *p* = 0.07) [21]. Rodrigo et al. have evaluated 310 adult patients receiving twice-daily TAC, in whom the presence of dnDSA was analyzed at one, three, and five years and around the sixth month before the last follow-up visit. Among 116 patients, 37.4% demonstrated CV greater than 30%, and 39 patients (12.6%) developed dnDSA. The variation coefficient greater than 30% (hazard ratio, 2.613; 95% confidence interval, 1.361–5.016; *p* = 0.004) was independently related to graft loss. Acute rejection, re-transplant, and CV greater than 30% (hazards ratio, 2.925; 95% confidence interval, 1.473–5.807; *p* = 0.002) were the only variables related to dnDSA development, as demonstrated by Cox regression analysis [19]. We have also evaluated values of a cumulative effect strength of triple-drug immunosuppressive protocol in the prospective patients. The cumulative Vasudev scores have been calculated at several time-points; however, at any point the scores were not significantly different between dnDSA-positive and dnDSA-negative patients. So far, Vasudev scores have been used in limited number of studies, thereby limiting the possibility to compare our results with other reports. The values of 12.0 ± 2.9 vs. 11.0 ± 3.0 and 7.5 ± 1.7 as 6.8 ± 2, respectively, have been reported at discharge and 1-year post- transplantation by Höcker et al., who used this parameter as a surrogate marker of cumulative strength of immunosuppression in pediatric kidney recipients, evaluated in terms of EBV infection risk. The value of this score was significantly higher (*p* = 0.009) in patients who developed EBV infection, and might reflect the adverse (infection-related) consequences of a high cumulative immunosuppressive load [16]. Basing on our results, we may suggest that the main immunosuppressive effect in prospectively evaluated patients was related to the tacrolimus concentration, as a cumulative score was similar in DSA producers and non-producers over time after transplantation. There is an ongoing discussion on the clinical relevance and cost-effectiveness of a close protocol monitoring of dnDSA generation in patients with different immunological risk. We have demonstrated that evaluation of dsDSA to the late, by-cause tested cases, even limited to patients with low immunological risk, was associated with high incidence of cAMR and inferior outcome in terms of graft survival within a further three years after diagnosis. On the other hand, regular screening for dnDSA performed every three months within the first two years after transplantation in patients with low immunological risk and low risk of non-adherence (expressed as low variability of trough concentration of calcineurin inhibitor, mainly tacrolimus), on triple immunosuppressive protocol without induction, appeared not to be justified due to low incidence of dnDSA detection, and good outcomes. Therefore, we consider that prospective screening for dnDSA in such patients should be limited to a regular annual test, with closer monitoring in symptomatic cases, once DSA have been identified. The expert consensus guidelines of the Transplantation Society state, that low-risk patients (not sensitized first transplantation) should be screened for DSA at least once 3 to 12 months after transplantation, with a kidney biopsy performed when DSA has been detected [6]. We recognize the limitations of this study related to relatively small number of patients and the inclusion of a retrospective group to some parts of the analysis. Limitation of the detailed statistical analysis in regard to the immunosuppression exposures, to a prospective group, made the analysis more reliable; however, it reduced the number of patients and relevant numerical parameters available for the evaluation. Therefore, the results of this study should be verified in more numerous prospective cohort. On the other hand, we were able to present the difference in the attitudes towards dnDSA screening over the years in a single transplant center. Despite ongoing discussion on the optimal and individualized threshold of the immunosuppression, the increased availability of dnDSA testing and more precise definitions of DSA specificity, related to eplet matching potentially might make this tool more effective [36].

## 5. Conclusions

In summary, we have found that in patients prospectively screened for dnDSA, the incidence of dnDSA increased regularly with time of follow-up, the presence of dnDSA was associated with the significant decrease of eGFR over time, and this was associated with lower exposure to tacrolimus (despite remaining within the therapeutic range of blood concentration). We did not confirm a significant meaning of the tacrolimus blood concentration variability, nor a value of cumulative (Vasudev) scores in this term.

## Figures and Tables

**Table 1:** Modified Vasudev immunosuppressive score [16].

Drug	Pediatric Score: Dose per Unit (mg/m^2^/d)	Immunosuppressive Unit
Tacrolimus (TAC)	1.2	1
Cyclosporin A (CsA)	58	1
Sirolimus (SRL)	1.2	1
Mycophenolate mofetil (MMF)	290	1
Azathioprine (AZA)	58	1
Prednisone (equivalent)	2.9	1

**Table 2:** Baseline characteristic of patients.

	Retrospective Group (*n* = 29) Median (Q1–Q3) ^#^ *n* (%)	Prospective Group (*n* = 38) Median (Q1–Q3) *n* (%)	*p*
Age, years	8.1 (5.0–9.9)	11.4 (8.0–14.5)	0.002
No. HLA-DR mismatches: 0/1/2	5/13/11 (17/45/38)	8/25/5 (21/66/13)	0.06
No. HLA-B mismatches: 0/1/2	1/15/13 (3/52/45)	2/19/17 (5/50/45)	1
No. HLA-A mismatches: 0/1/2	3/14/12 (10/48/41)	4/19/15 (11/50/39)	1
HLA A+B+DR mismatches	4 (3–5)	4 (3–4)	0.3
Maintenance immunosuppression			
TAC	25 (86.2)	31 (81.6)	0.75
CsA	7 (24.1)	7 (18.4)	0.57
MMF	25 (86.2)	37 (97.4)	0.16
Pred	29 (100)	38 (100)	N/A
Baseline eGFR (mL/min/1.73 m^2^),	72.8	76.4	0.27
No. of patients with kidney biopsies	(53.6–93.1)	(70.3–99.5)	
	23 (79.3)	10 (26.3)	<0.0001
Follow-up, years	8 (6–11)	2 (2–2)	<0.0001
Incidence of dnDSA (%) after transplantation overall	100	21	N/A
at 3 months	N/A	8	N/A
at 6 months	N/A	11	N/A
at 12 months	N/A	16	N/A
at 2 years	N/A	21	N/A

^#^ Q1, lower quartile; Q3, upper quartile; N/A, not applicable.

**Table 3:** Kidney function (eGFR), incidence of acute rejection and cAMR, in the retrospective and the prospective groups.

Variable	Group		*p*
	Retro Median (Q1–Q3) *n* (%)	Pro Median (Q1–Q3) *n* (%)	
Incidence of dnDSA	29 (100)	8 (21)	<0.0001
eGFR at detection of dnDSA, mL/min/1.73 m^2^	41.0 (28.7–57.2)	85.0 (55.0–92.5)	0.004
Incidence of biopsy proven rejection	19 (66)	7 (18)	<0.0001
Incidence of graft loss in cAMR	12 (41)	0	<0.0001

**Table 4:** Median values and variation of TAC C_0_ in all consecutive evaluations between 1st and 24th month after transplantation in the prospective group.

Parameter	dnDSA (−)	dnDSA (+)	*p*
TAC C_0_ (ng/mL), median, (Q1–Q3)	7.9 (6.5–10.3)	7.1 (5.9–8.5)	0.0088 ^
TAC C_0_ coefficient of variation (%), median (Q1–Q3)	31 (23–39)	29 (17–37)	0.56

^ repeated measures linear mixed model.

**Table 5:** Median values of cumulative Vasudev score between 1st and 24th month after transplantation in the prospective group.

Vasudev Score, Median, (Q1–Q3)	dnDSA (−)	ndDSA (+)	*p*
1 month after transplantation	10.6 (8.6–12.5)	10.5 (8.4–11.0)	NS ^
12 months after transplantation	6.3 (5.5–7.0)	6.1 (5.4–6.6)	NS ^
2 years after transplantation	5.3 (4.1–6.0)	4.1 (3.8–5.9)	NS ^

^ repeated measures linear mixed model. NS—not-significant.

## Data Availability

Most of the relevant data are presented in the manuscript. The source data are archived in hospital medical records and internal hospital databases. The access to the source data is limited by in-hospital system to the authorized medical staff representatives.
